# PREPARING YOUR AGEC SELF-STUDY

**DOI:** 10.1093/geroni/igz038.1471

**Published:** 2019-11-08

**Authors:** Donna Weinreich, Donna Schafer

**Affiliations:** 1 Western Michigan University, Kalamazoo, Michigan, United States; 2 National Association for Professional Gerontologists, Healdsburg, California, United States

## Abstract

Section VI of the AGEC Handbook provides guidelines for writing the self-study. This Handbook section is central to the accreditation review process because it provides information about how your program demonstrates that accreditation standards have been met/exceeded. It also specifies preparatory work that programs should undertake prior to applying for accreditation. Based on insights gained from the first round of AGEC accreditation reviews at the master’s, baccalaureate and associate levels, Section VI of the Handbook has been revised to clarify expectations about the self-study. Specifically, greater emphasis has been placed on 1) following the Standards outline in Section V of the Handbook, 2) insuring that all relevant information is contained in the self-study and, 3) requiring that the complete document, including appendices, is submitted well in advance of the site visit. This presentation describes the content and revisions in Section VI, as well as the process for submitting the self-study.

